# An Unexpected Association of Primary Biliary Cholangitis in a Male Patient With Autosomal Dominant Polycystic Kidney Disease

**DOI:** 10.7759/cureus.39517

**Published:** 2023-05-26

**Authors:** Patricio A Jaramillo, Sabina Ibrahimli, Javier Castells

**Affiliations:** 1 General Practice, Universidad Estatal de Guayaquil, Machala, ECU; 2 Cardiology, First Moscow State Medical University named after Sechenov, Moscow, RUS; 3 Medicine, Universidad Católica de Santiago de Guayaquil, Guayaquil, ECU

**Keywords:** complication, men, clinical presentation, pbc, primary biliary cholangitis, autosomal dominant polycystic kidney disease, cholangitis

## Abstract

Autosomal dominant polycystic kidney disease (ADPKD) is a genetic disorder that leads to various long-term complications. However, we are describing an association between this patient’s condition and primary biliary cholangitis (PBC). A 55-year-old male patient presented to our clinic with PBC, demonstrating the importance of how PBC can be clinically asymptomatic and the importance of the criteria given to diagnose the disease. We encourage physicians to periodically check all ADPKD patients to guard against asymptomatic problems that might endanger their health in the future.

## Introduction

A chronic, seropositive, inflammatory, and cholestatic liver illness known as primary biliary cholangitis (PBC) progresses at a variable rate to biliary cirrhosis [1]. PBC is most prevalent in middle-aged females but can also affect young females and males. PBC is recognized as an autoimmune disorder because of the development of illness-specific autoantibodies such as antimitochondrial antibodies (AMAs), the significant infiltration of mononuclear cells into the bile ducts, and the broad spectrum of comorbid autoimmune conditions [2]. PBC is characterized histologically by the degeneration and necrosis of intrahepatic biliary epithelial cells surrounded by considerable infiltration of mononuclear cells, causing destructive changes and major loss of small- or medium-sized bile ducts. We present a case of PBC in an asymptomatic patient with a history of several comorbidities who presented with liver function test (LFT) alterations, meeting two of the three criteria needed for diagnosing PBC. Ten months before presenting to our clinic, the patient was diagnosed with autosomal dominant polycystic kidney disease (ADPKD), an autoimmune disease usually affecting several systems; this diagnosis is a rare association with ADPKD. Diagnostic procedures were performed on the patient to rule out the most frequently related causes of PBC, and as a result of this exclusion, we came to the conclusion that his PBC was secondary to ADPKD.

## Case presentation

An asymptomatic 55-year-old male with a history of colon polyps of the ascending colon, asthma, hypothyroidism, essential hypertension, diabetes mellitus type 2, hypercholesterolemia, and ADPKD was diagnosed in April 2022. The patient had no history of using illegal substances or alcohol, nor did he smoke. The patient’s vital signs were within the normal range, except for a BMI of 33. The patient’s primary care physician (PCP) referred him for a review of abnormal liver function tests. Laboratory tests are shown in Table 1.

**Table 1:** Autoimmune, comprehensive, and tumor marker panel

Test Name	Out of Range	Reference Range
Alkaline phosphatase	253 U/L	35-144 U/L
Alkaline phosphatase liver isoenzymes	74%	25-69%
Alkaline phosphatase bone isoenzymes	23%	28-66%
Alanine transaminase	69 U/L	9-46 U/L
Aspartate aminotransferase	65 U/L	10-35 U/L
Glucose	46 mg/dL	65-99 mg/dL
Thyroid-stimulating hormone	11.2 mIU/L	0.40-4.50 mIU/L
Direct bilirubin	0.3 mg/dL	0.2-1.2 mg/dL
Alpha fetoprotein	2.43 ng/mL	0-40 ng/mL
Antibody SS-B (La)	Negative	Negative
Antibody SS-A (Ro)	Negative	Negative
Tumor marker Ca 19-9	11 U/mL	<34 U/mL
Antinuclear antibody titers	≥1:1280	Negative titer
Actin (smooth muscle) antibody (IgG)	<20 U	<20 U
Mitochondrial antibody titer	1:160 titer	<1:20 titer
Gamma-glutamyl transferase	865 U/L	3-85 U/L
Thyroid peroxidase antibodies	10 IU/mL	<9 IU/mL
Hepatitis A virus IgM antibodies	Non-reactive	Non-reactive
Hepatitis B virus surface antibody	Non-reactive	Non-reactive
Hepatitis B virus core	Non-reactive	Non-reactive
Hepatitis B virus e antigen	Non-reactive	Non-reactive
Hepatitis C virus IgG antibodies	Non-reactive	Non-reactive

**Figure 1:**
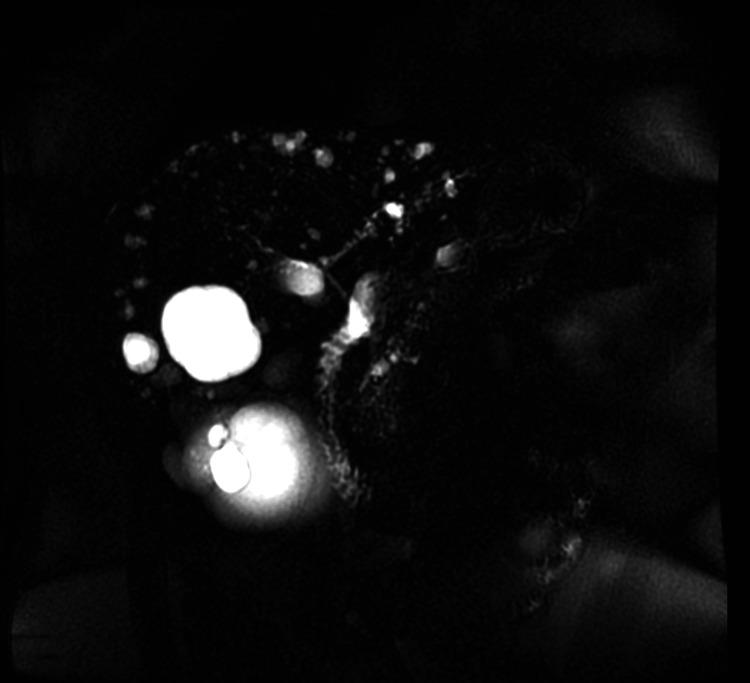
Magnetic resonance cholangiopancreatography showing multiple liver cysts

**Figure 2:**
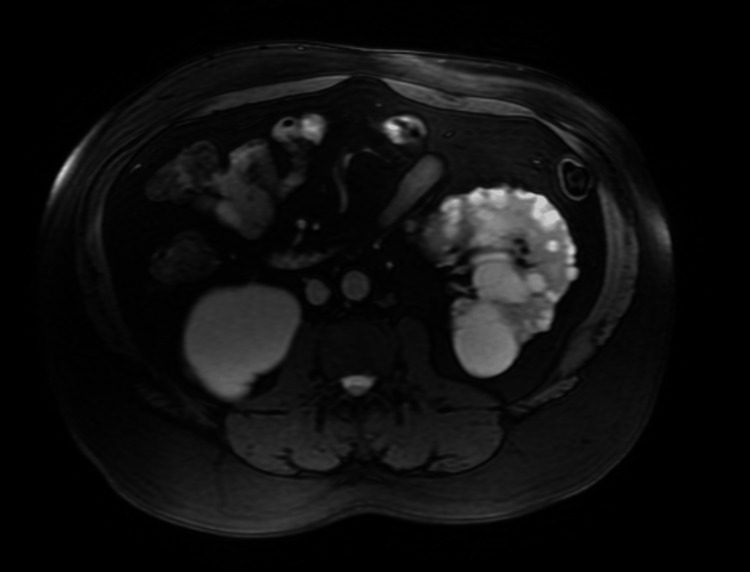
Magnetic resonance cholangiopancreatography showing multiple left kidney cysts

Imagenology provided by his PCP showed an MRI of the abdomen without contrast that showed innumerable small cystic lesions scattered throughout the pancreas, with the largest seen in the pancreatic head. There were also innumerable simple and mildly complex renal cysts, multiple hepatic cysts, and possible biliary hamartomas. A CT scan showed an area of parenchymal prominence at the proximal pancreatic body, and the kidneys were asymmetric in size. Multiple simple and mildly complex cysts were seen bilaterally, suggesting polycystic kidney disease (Figures 1-2). The most recent esophagogastroduodenoscopy done in 2022 did not demonstrate any abnormalities. He takes hydrochlorothiazide 12.5 mg orally (PO), simvastatin 40 mg, levothyroxine 100 mcg, fluticasone propionate 50 mcg, albuterol 0.63 mg/3 mL, and metformin 500 mg. After excluding any common disease associated with this patient’s laboratory abnormalities, he was diagnosed with PBC, possibly associated with ADPKD. The patient is being treated with high-dose ursodeoxycholic acid (UDCA) at 15 mg/kg twice a day and under lifelong follow-up to assess therapeutic response and manage symptoms if any develop while being mindful of the threat of cirrhosis or hepatocellular carcinoma.

**Figure 3:**
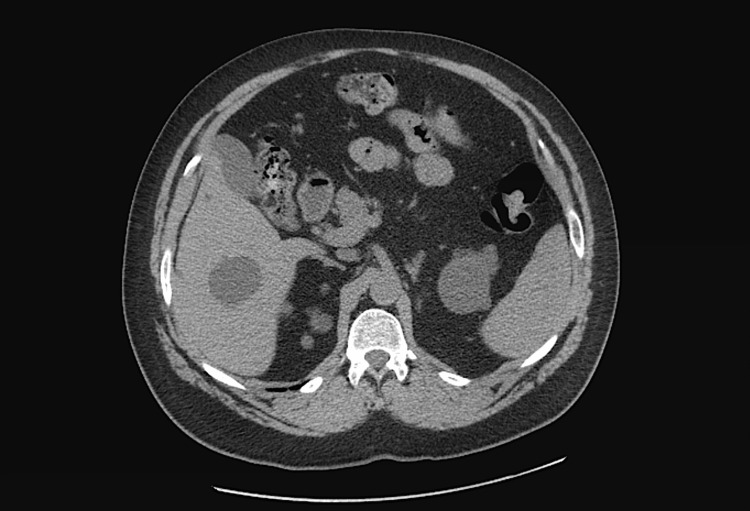
Abdominal CT scan showing hepatic cyst

**Figure 4:**
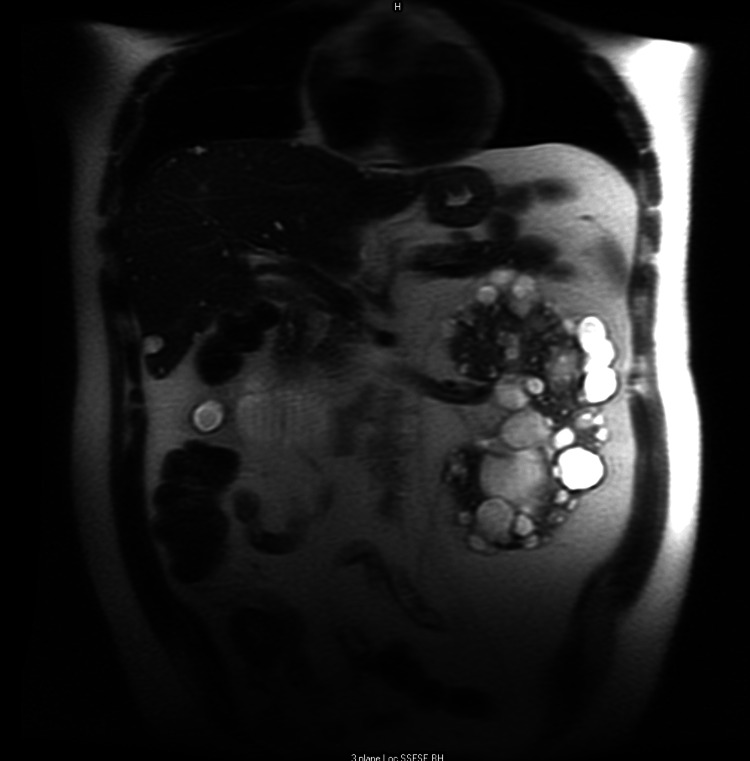
Magnetic resonance cholangiopancreatography showing multiple left kidney and liver cysts

Figure 3 shows abdominal CT scan. Figure 4 depicts magnetic resonance cholangiopancreatography showing multiple left kidney and liver cysts.

## Discussion

In this case report, an asymptomatic patient with a recent ADPKD diagnosis showed up for medical consultation regarding abnormal LFTs. He presented two of the three criteria for being diagnosed with PBC, and the attending doctor supported the suspicion that the PBC may have been secondary to ADPKD, which he treated with UDCA. The patient’s nephrologist should closely monitor his ADPKD to avoid future complications. This new presentation of PBC secondary to ADPKD demonstrates that the aforementioned condition can affect the biliary tract. Therefore, we advise special attention during the routine laboratory test review to take priority when taking clinical, personal, social, and family histories to detect the PBC presentation form of the disease. A family history of PBC, a history of urinary or vaginal infections, and the regular use of nail polish or hair dye have all been linked to the disease, which is not the case in our patient. However, the patient presented with comorbidities and other autoimmune diseases, including diabetes mellitus type 2 and hypothyroidism [3]. Concerning our investigation, the patient satisfied two out of the three criteria to diagnose PBC: persistently elevated serum alkaline phosphatase and serum AMA. Therefore, the criterion of liver histology was not evaluated to avoid an invasive procedure [3,4]. The patient will have a liver function test evaluation every three to six months while on UDCA. UDCA is an endogenous hydrophilic bile acid that acts as an apoptosis blocker by supporting the mitochondrial membrane or affecting the expression of specific upstream targets [5]. A decrease in liver function tests was seen in those who took UDCA, leading to many clinical studies investigating UDCA’s effects on PBC [5].

## Conclusions

The patient in this instance acquired PBC, and after ruling out the most frequent conditions that are connected with it, we came to the conclusion that ADPKD may be related to this diagnosis. In order to rule out any shared associations with the condition, we gathered personal, social, familial, surgical, and pharmaceutical histories. The patient was asymptomatic, presenting with abnormal liver function and newly diagnosed ADPKD and fulfilling the criteria for PBC diagnosis. In his differential diagnosis, he has other autoimmune comorbidities diagnosed more than five years ago, such as diabetes mellitus type 2 and hypothyroidism, which were ruled out as associated with his diagnosis due to his adherence to treatment for these conditions.
